# Bimetallic NiCo Nanoparticles Embedded in Organic Group Functionalized Mesoporous Silica for Efficient Hydrogen Production from Ammonia Borane Hydrolysis

**DOI:** 10.3390/nano14221818

**Published:** 2024-11-13

**Authors:** Juti Rani Deka, Diganta Saikia, Ning-Fang Lu, Chieh-Yu Chen, Hsien-Ming Kao, Yung-Chin Yang

**Affiliations:** 1 Institute of Materials Science and Engineering, National Taipei University of Technology, Taipei 106344, Taiwan; juti.deka@gmail.com; 2 Department of Chemistry, National Central University, Zhongli 320317, Taiwan; digantas@gmail.com (D.S.); foxflowerlnf@gmail.com (N.-F.L.); scwhya1111756@gmail.com (C.-Y.C.)

**Keywords:** bimetallic NiCo nanoparticles, mesoporous silica, nanocatalyst, ammonia borane hydrolysis, hydrogen generation

## Abstract

In this study, bimetallic NiCo nanoparticles (NPs) were encapsulated within the mesopores of carboxylic acid functionalized mesoporous silica (CMS) through the chemical reduction approach. Both NaBH_4_ and NH_3_BH_3_ were used as reducing agents to reduce the metal ions simultaneously. The resulting composite was used as a catalyst for hydrolysis of ammonia borane (NH_3_BH_3_, AB) to produce H_2_. The bimetallic NiCo NPs supported on carboxylic group functionalized mesoporous silica, referred to as Ni_x_Co_100−x_@CMS, exhibited significantly higher catalytic activity for AB hydrolysis compared to their monometallic counterparts. The remarkable activity of Ni_x_Co_100−x_@CMS could be ascribed to the synergistic contributions of Ni and Co, redox reaction during the hydrolysis, and the fine-tuned electronic structure. The catalytic performance of the Ni_x_Co_100−x_@CMS nanocatalyst was observed to be dependent on the composition of Ni and Co. Among all the compositions investigated, Ni_40_Co_60_@CMS demonstrated the highest catalytic activity, with a turn over frequency (TOF) of 18.95 mol_H2_min^−1^mol_catalyst_^−1^ and H_2_ production rate of 8.0 L min^−1^g^−1^. The activity of Ni_40_Co_60_@CMS was approximately three times greater than that of Ni@CMS and about two times that of Co@CMS. The superior activity of Ni_40_Co_60_@CMS was attributed to its finely-tuned electronic structure, resulting from the electron transfer of Ni to Co. Furthermore, the nanocatalyst exhibited excellent durability, as the carboxylate group in the support provided a strong metal–support interaction, securely anchoring the NPs within the mesopores, preventing both agglomeration and leakage.

## 1. Introduction

Hydrogen, a clean and renewable energy carrier, is regarded as one of the most promising new energy sources to meet the growing demand for sustainable and environmentally friendly energy solutions [1]. Currently, most hydrogen is produced through processes such as steam reforming, partial oxidation, or gasification processes of fossil fuels [2,3]. During the steam reforming of natural gas, steam reacts with natural gas at high temperatures (700–1100 °C) to produce H_2_, CO_2_, and CO. The water–gas shift reaction, where CO reacts with steam to produce H_2_ and CO_2_, occurs simultaneously with the reforming process. H_2_ production through water electrolysis, in which water is separated into H_2_ and O_2_ using electricity, is recognized as a promising renewable resource [4]. H_2_ can also be produced through gasification of coal or biomass, where coal reacts with steam, air, or O_2_, releasing gases such as CO, CO_2_, H_2_, H_2_O (vapor), and CH_4_. Depending on the gasification system, a residue, such as ash or molten slag, is also accumulated [5]. The most cost-effective and environmentally friendly way to produce green fuels for energy is likely through water splitting to generate H_2_ and O_2_ [6]. The safe storage and transport of H_2_ remain the most significant challenges for advancing towards H_2_ energy technology. Consequently, significant research is focused on producing hydrogen from various portable hydrogen sources [7,8]. Among these, hydrogen storage materials such as organic compounds, borohydrides, and metal hydrides [9,10,11] have gained increasing attention in recent years. Ammonia borane (NH_3_BH_3_, AB), a solid non-toxic hydrogen storage material, offers several advantages such as high hydrogen content (19.6 wt.%), low molecular weight (30.86 g mol^−1^), high volumetric hydrogen density (0.145 kg H_2_ L^−1^), and high stability [12,13,14]. It can produce clean H_2_ through dehydrogenation, hydrolysis, and methanolysis reactions. Catalytic hydrolysis of NH_3_BH_3_ with appropriate catalysts is considered as an efficient method for rapid H_2_ generation. Along with a substantial amount of H_2_, hydrolysis produces environmentally friendly byproducts such as H_3_BO_3_, NaBO_2_, and BO_2_ [15]. However, efficient and cost-effective use of ammonia borane for H_2_ generation continues to be significantly challenging, largely due to the lack of a stable and efficient catalyst, which impedes its practical applications. While noble metal catalysts, such as Pt, Pd, Ru, and Rh [16,17,18,19] have been traditionally used as catalysts due to their higher activity for H_2_ generation from NH_3_BH_3_ hydrolysis, their scarcity and high cost inevitably limit their large-scale applications. The recent advancements made in the synthesis procedures, such as co-precipitation, sol-gel, and chemical reduction, have enabled the production of a range of highly efficient non-noble metal-based catalysts such as Co, Ni, Cu, and Fe for successful H_2_ generation from AB hydrolysis [20,21,22,23,24]. These non-noble metals are abundant in nature and significantly cheaper as compared to noble metals. Their natural availability greatly reduces the environmental risks linked to mining and processing, making them a greener choice for large-scale hydrogen production. Additionally, their excellent recyclability minimizes waste, helping to lower overall environmental pollution. The use of non-noble metal catalysts has led to significant improvements in terms of performance, cost-effectiveness, and environmental sustainability. Xu et al. have successfully developed non noble metal based Co, Ni, and Cu catalysts and used them for hydrogen generation from ammonia borane. The catalysts demonstrated high activity in the hydrolysis of NH_3_BH_3_, releasing 3 moles of hydrogen per mole of NH_3_BH_3_, with purity sufficient for direct use in proton exchange membrane (PEM) fuel cells [25]. Fang et al. encapsulated ultra-small Co nanoclusters in soluble porous coordination cages (Co NCs@PCC), wherein the particle size of Co NCs was only 2.5 nm and PCC could very well stabilize and disperse Co NCs. The catalytic performance of the catalysts could reach the level of noble metals [26]. Sun’s group reported 3.2 nm monodispersed Ni nanoparticles (NPs) with a low activation energy, high TOF, and excellent reusability, which were comparable to that of Pt-based catalysts [27]. The easy preparation and high catalytic performance revealed their promising application possibility for the development of an efficient and portable hydrogen production system. Hu et al. prepared Co NPs supported on polyethylenimine and graphene oxide and proved that the supported catalyst presented extremely high dehydrogenation activity under an atmospheric condition, which was also close to that of noble metal-based catalysts [28]. Li et al. developed low-cost CuFeCo@MIL-101 as an efficient catalyst for catalytic hydrolysis of ammonia borane, which showed potential for application in the field of hydrogen storage [29]. The implementation of a portable power supply system using liquid hydrogen storage technology is highly significant both economically and scientifically. This technology is anticipated to have applications across diverse fields, including hydrogen-powered drones and large hydrogen-powered buses. However, monometallic nanocatalysts often have low catalytic activity and poor stability as they are prone to deactivation due to aggregation, poisoning, and oxidation during synthesis and catalysis. In contrast, bimetallic nanocatalysts are catalytically more active as the combination of two metals can create new electronic or structural properties that improve the catalytic performance, efficiency, and stability [30]. The incorporation of the second metal during the synthesis of bimetallic NPs can restrict the overgrowth and aggregation of the active components by altering the nucleation and growth kinetics, which is important for the enhancement of catalytic activity [31,32,33,34]. Co-based non-noble metal bimetallic nanocatalysts are the most promising heterogeneous catalysts as Co is relatively cheap, readily available in nature, and highly active [35,36]. Furthermore, Co-based catalysts exhibit better durability than other catalysts and can maintain their catalytic activity for extended periods, making them a preferred choice for AB hydrolysis. Even though bimetallic NPs are less prone to aggregation compared to monometallic ones, they can still undergo aggregation under certain conditions such as high temperature and the presence of reactive species, which greatly influences the catalytic activity. Immobilizing bimetallic NPs on support materials with a high surface area, including metal oxides [37], carbon materials [14,38], and porous materials [30,39], can prevent their potential aggregation during preparation and catalysis and thus can enhance the long-term stability. Furthermore, porous materials like metal organic frameworks (MOFs), ordered mesoporous silicas (OMS), and mesoporous carbons can provide precise control of bimetallic NPs with a narrow size distribution. OMS with a large surface area and ordered mesopores such as SBA-15 [40,41,42] and MCM-41 [43] have been extensively utilized as supports to immobilize bimetallic NPs. The supported metal NPs serve as an effective catalyst for H_2_ generation from ammonia borane due to their stable chemical properties. The confinement effect of the mesopores of OMS, especially with 3-dimensional (3D) pore arrangements, such as SBA-16 and FDU-12, are suited for immobilization of metal NPs since their multidirectional mesoporous channels greatly reduce the risk of pore blockage [44,45]. The interconnected mesoporous architecture enables the efficient transport of reactant and product molecules during the catalytic reaction. Research has shown that the surface properties of OMS significantly influence the distribution and loading of metal NPs and hence the catalytic activity. In general, OMS with silanol groups on the surface are not effective for controlling NPs aggregation because of the low electrostatic affinity between the silanols and metal ions. The surface of OMS are therefore modified by incorporating organic functional groups including thiol (-SH) [46], carboxylic acid (-COOH) [47], and amino (-NH_2_) groups [48] to intensify the attraction between the support and the metal precursor. These functional groups, which are dispersed homogenously, serve as anchoring sites for metal precursors, thus controlling the nucleation and growth of the metal NPs. This precise control over nucleation sites helps in controlling both the size and shape of the NPs, as growth is localized around the functional groups. Additionally, surface modification improves the distribution of metal NPs on the support, preventing aggregations and thereby maintaining a high surface area, essential for efficient catalysis. The acidic or basic nature of the support can also be tuned by incorporating appropriate functional groups, with acidic functionalization reported to enhance reactions involving protonation or activation of the reactant. Moreover, the functional groups on the surface of the pore strengthen the interaction between the metal and the support, modifying the electronic properties of the metal. The strong metal–support interaction enhances the catalytic activity by improving the adsorption or activation of the reactants. The functional groups on the surface of the pore not only assist in the uniform distribution of the metal ions in the pores, but also subsequently generate highly dispersed metal NPs. The functional groups on the surface of the pore can thus create strong metal−support interactions, which can further enhance the catalytic performance.

Among various transition metal-based bimetallic nanocatalysts, Co-based nanocatalysts, particularly those based on NiCo NPs, have received special attention for H_2_ production from AB hydrolysis due to their excellent catalytic activity, stability, and low cost [49,50,51,52]. While various supporting materials such as graphene oxide (GO), reduced graphene oxide (RGO), carbon nanotubes, Al_2_O_3_, and porous carbon have been used to immobilize bimetallic NiCo NPs for AB hydrolysis to generate hydrogen [33,49,53,54,55,56,57,58,59], the utilization of mesoporous silicas as supports for immobilizing NiCo NPs and their applications as catalysts for AB hydrolysis has not yet been reported, to the best of our knowledge. Our previous study demonstrated that bimetallic CoCu NPs supported on mesoporous silicas exhibited better catalytic performance compared to the monometallic counterparts Co or Cu [60]. However, between Cu and Ni, Cu is catalytically less active in the hydrogen generation reaction compared to Ni. During the hydrolysis of ammonia borane using bimetallic CoCu NPs, Cu does not significantly contribute to dehydrogenation, leaving the catalytic burden mainly on Co. On the other hand, both Ni and Co have strong catalytic properties in hydrogen-related reactions. The Ni component in bimetallic NPs aids in the breakdown of AB, while Co enhances the reaction kinetics. Given the advantages of bimetallic NiCo NPs as a catalyst for AB hydrolysis, exploring the catalytic behavior of NiCo supported on mesoporous silicas would be of great interest.

In this study, carboxylic acid functionalized mesoporous silica was utilized as a support for immobilizing bimetallic NiCo NPs with various compositions, which were then used as catalysts for efficient H_2_ generation from AB hydrolysis. The carboxylic group was selected among various organic functional groups for surface functionalization of the mesoporous silica support due to its ability to form strong coordination bonds with metal ions, facilitating a supporting environment for the growth of metal NPs within the mesoporous silica support. Carboxylic group functionalized mesoporous silica was synthesized via co-condensation of tetraethyl orthosilicate (TEOS) and carboxyethylsilanetriol sodium salt (CES) in an acidic medium. The surface charge of the carboxylated mesoporous silica was deprotonated to carboxylate (-COO^−^) to enhance the interactions with metallic ions. During the synthesis of bimetallic NiCo NPs, positively charged Ni^+2^ and Co^2+^ ions easily diffused into the interior of the mesopores due to the strong electrostatic interactions with the negatively charged carboxylate groups within the mesopores. The embedded Ni^+2^ and Co^2+^ ions were simultaneously reduced using two reductants, NaBH_4_ and NH_3_BH_3_, together to form homogeneously dispersed bimetallic NiCo NPs. The inclusion of the comparatively gentler reducing agent NH_3_BH_3_, along with NaBH_4_, slowed down the rapid reduction of the metal ions and regulated the growth of the NPs. The nanocatalysts thus obtained were employed as catalysts for hydrogen production through the hydrolysis of ammonia borane. The effects of various factors such as the composition of both the metals in the composite, metal concentration, and temperature on the catalytic activity were studied. The reusability of the bimetallic NiCo nanocomposite catalyst was also tested to evaluate its potential for practical applications.

## 2. Materials and Methods

### 2.1. Synthesis of NiCo Nanoparticles Supported on Organic Group Functionalized Mesoporous Silica

Organic group functionalized mesoporous silica supported bimetallic NiCo NPs were synthesized by co-impregnation of nickel (II) nitrate hexahydrate (Ni(NO_3_)_2_·6H_2_O ((Alfa Aesar, Heysham, UK) and cobalt nitrate hexahydrate (Co(NO_3_)_2_·6H_2_O, Alfa Aesar) precursors within the mesopores, followed by simultaneous reduction with two reductants, NaBH_4_ (Aldrich, St. Louis, MO, USA) and NH_3_BH_3_ (AK Scientific, Union City, CA, USA), simultaneously. In a typical synthesis, carboxylic acid functionalized mesoporous silica, referred to as CMS, was first prepared as the support material by co-condensing tetraethyl orthosilicate (TEOS, Aldrich) with carboxyethylsilanetriol sodium salt (CES, Gelest, Morrisville, PA, USA) in the presence of poly(ethylene oxide)-poly(propylene oxide)-poly (ethylene oxide) (Pluronic 123, P123) and Pluronic^®^F-127 (Aldrich). A detailed description of the synthesis process of CMS was given elsewhere [60]. Next, 0.1 g of CMS was suspended in a mixed solution consisting of 10 mL of Co(NO_3_)_2_·6H_2_O (0.01 M) and 10 mL of Ni(NO_3_)_2_·6H_2_O (0.01 M), stirred for 10 min, and then subjected to ultrasonication for one hour. Separately, another solution was prepared by mixing NaBH_4_ and NH_3_BH_3_, which was then added dropwise to the previously prepared mixed metals precursor solution. The mixture was stirred continuously until the bubbling stopped. The solid was recovered through centrifugation, washed with de-ionized water, and dried. The molar ratios of the various components in the reaction mixture were as follows: 1 (Co(NO_3_)_2_·6H_2_O or/and Ni(NO_3_)_2_·6H_2_O):54 NH_3_BH_3_:8.8 NaBH_4_:18,600 H_2_O. During the preparation, the overall volume of the metal precursors was fixed at 10 mL, irrespective of the individual quantities of each component. The synthesized nanocomposite was named as Ni_x_Co_100−x_@CMS, where *x* represents the mass percentage of Ni in the composite. An *x* value of 100 corresponds to the presence of Ni only, referred to as monometallic Ni@CMS, while an *x* value of 0 corresponds to the presence of Co only, denoted as monometallic Co@CMS.

### 2.2. Characterizations

The structural order and successful incorporation of the organic groups in the CMS support were studied using the Wiggler-A beam line (λ = 0.133367 nm) in the National Synchrotron Radiation Research Center (NSSRC) in Taiwan and a JASCO FTIR-4100 (JSCO, Tokyo, Japan), respectively. The crystallinity and the phase structure of Ni_x_Co_100−x_@CMS were analyzed using an X-ray diffractometer (Lab-x XRD-6000 SHIMADZU, Kyoto, Japan) and transmission electron microscopy (TEM, JEOL JEM2100, Tokyo, Japan). The textural properties of the composites were explored from the N_2_ adsorption–desorption isotherms with an Autosorb iQ2 (Auton Paar, Graz, Austria) at 77 K. The Barrett–Joyner–Halenda (BJH) method was employed to estimate the pore size and pore volume. The chemical state of Ni_x_Co_100−x_@CMS was analyzed using a Thermo VG (Thermo Fisher Scientific, Waltham, MA, USA) scientific sigma probe X-ray photoelectron spectrometer. The amount of metal present in the Ni_x_Co_100−x_@CMS nanocomposite was estimated using a Jarrell-Ash, Bristol, UK, ICAP 9000, an inductively coupled plasma optical emission spectrometer (ICP-OES). The zeta potential was measured using a Zetasizer Nano-ZS90 laser particle analyzer (Malvern Instruments, Malvern, UK) at 25 °C.

### 2.3. Hydrolysis of Ammonia Borane with Ni_x_Co_100−x_@CMS Catalysts

The activity of the Ni_x_Co_100−x_@CMS catalysts toward the hydrolysis of ammonia borane was evaluated via real time monitoring of the H_2_ generation volume in a water-filled measuring cylinder system. In the typical experimental procedure, 5 mL of distilled water was mixed with 10 mg of Ni_x_Co_100−x_@CMS catalyst in a two-necked round-bottom flask and sonicated for 5 min in an ultrasonic bath. The flask was then placed in a water bath, which was thermostated to 30 °C and stirred continuously. A suitable amount of ammonia borane (0.33 wt.%) was then added to the flask, which was connected to a water-filled measuring cylinder. The catalyst to NH_3_BH_3_ molar ratio was kept as ~0.05. The volume of H_2_ generation was directly determined from the volume of displaced water level in the measuring cylinder due to the released H_2_. The change in volume was recorded every 30 s until the bubbles stopped forming. The reaction temperature was fluctuated within the range 30 to 55 °C to investigate the temperature effect and compute the energy of activation.

Reusability of the catalysts was assessed by conducting the AB hydrolysis experiments five times using the same catalyst. After each run, the catalyst was retrieved from the aqueous solution through centrifugation, rinsed with water, and subsequently dried for 24 h.

## 3. Results and Discussion

### 3.1. Structural and Morphological Characterizations of Ni_x_Co_100−x_@CMS

**Figure 1:**
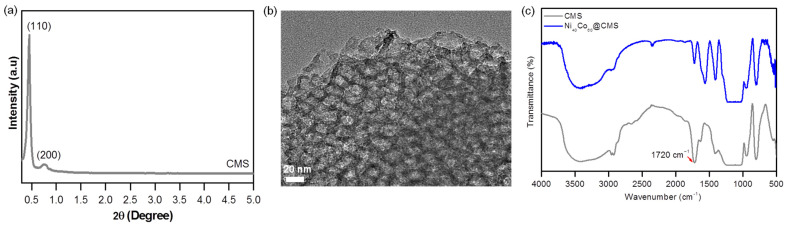
(**a**) SAXRD and (**b**) TEM image of CMS, (**c**) FTIR spectra of CMS and Ni_40_Co_60_@CMS.

Since the development of highly ordered mesopores and the presence of organic groups have been acknowledged as vital factors that influence the size and distribution of metal NPs when mesoporous silicas are utilized as supports, it is therefore important to verify the mesoporosity and successful incorporation of carboxylic acid groups into the mesoporous silica framework. The small angle X-ray diffraction (SAXRD) pattern of CMS, shown in Figure 1a, revealed two distinct peaks within the 2θ range of 0.5 to 1.5°, corresponding to the (110) and (200) reflections of the body-centered cubic (bcc) structure, which are similar to that observed for mesoporous silica SBA-16 [61]. The occurrence of the strong diffraction peak of the (110) reflection at 2θ < 0.5° indicated the development of well-ordered cubic mesopores in the CMS, which can also be realized from the transmission electron microscope (TEM) image shown in Figure 1b. The successful incorporation of the –COOH functional group into the mesopores of the CMS was confirmed by the incidence of an intense band at 1720 cm^−1^ [62,63] in the FTIR spectrum of the CMS, as shown in Figure 1c. The acid-base profile of the support was determined through titration using 0.01 M aqueous NaOH solution. The plot of the pH versus the amount of NaOH added is shown in Figure S1a (Supplementary Materials). The total acidic capacity of CMS was estimated to be 1.0 mmol g^−1^. The zeta potential was measured to evaluate the charge density in the CMS and was determined to be −3.8, −23, −28.8, and −43 mV at pH 3, 5, 7, and 9, respectively, as shown in Figure S1b. The incorporation of –COOH groups provided more negative charges on the support surface.

**Figure 2:**
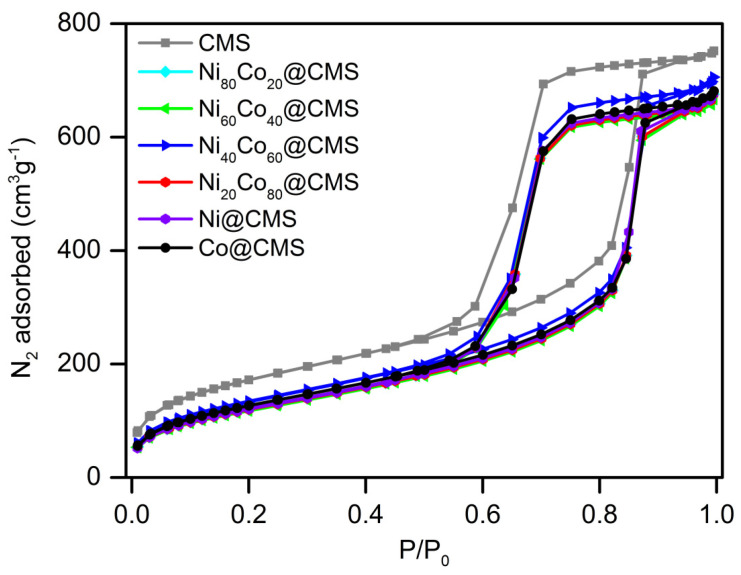
N_2_ adsorption-desorption isotherms of CMS, Ni@CMS, Ni_80_Co_20_@CMS, Ni_60_Co_40_@CMS, Ni_40_Co_60_@CMS, Ni_20_Co_80_@CMS, and Co@CMS.

**Figure 3:**
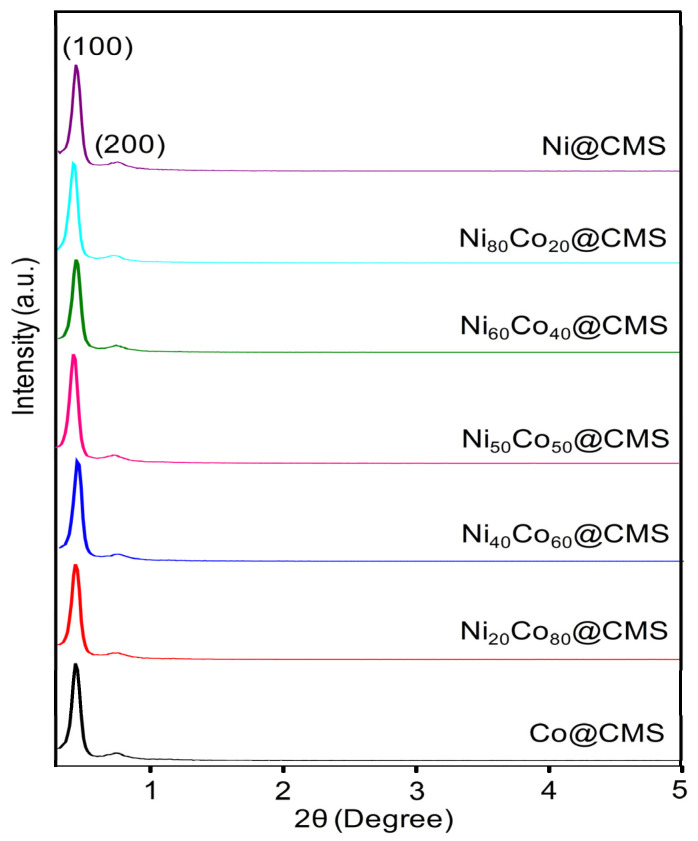
SAXRD patterns of Ni@CMS, Ni_80_Co_20_@CMS, Ni_60_Co_40_@CMS, Ni_50_Co_50_@CMS, Ni_40_Co_60_@CMS, Ni_20_Co_80_@CMS, and Co@CMS.

**Figure 4:**
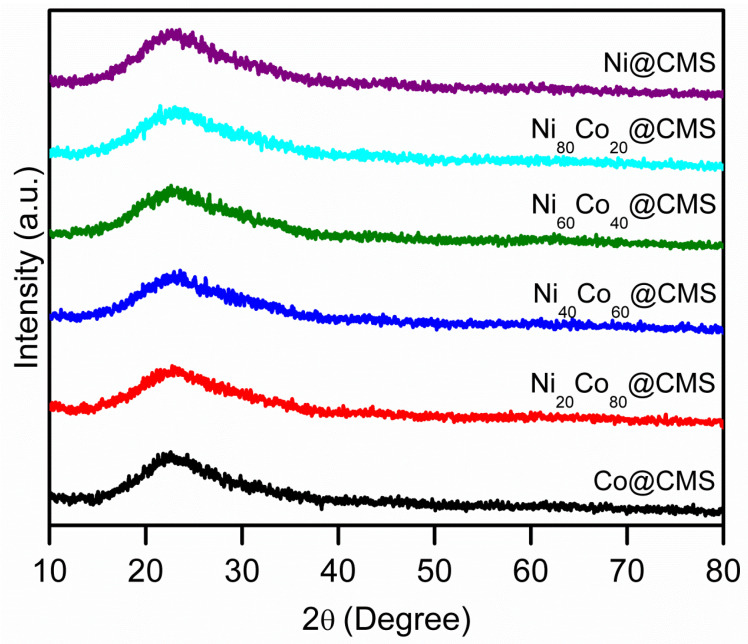
XRD patterns of Ni@CMS, Ni_80_Co_20_@CMS, Ni_60_Co_40_@CMS, Ni_40_Co_60_@CMS, Ni_20_Co_80_@CMS, and Co@CMS.

The N_2_ adsorption–desorption measurements shown in Figure 2 indicate a large specific surface area of CMS which assists in anchoring highly dispersed metal NPs. However, the BET surface area, pore size, and average pore volume of Ni_x_Co_100−x_@CMS were lesser than the CMS, as seen in Table 1, which hints at the successful incorporation of metal NPs into the mesopores of CMS. However, no significant change in the specific surface area was observed for all the CMS-supported metal NPs, which can be attributed to the homogeneous distribution of the metal NPs within the support. The N_2_ adsorption–desorption isotherms of Ni_x_Co_100−x_@CMS (x = 0, 20, 40, 60, 80, 100) shown in Figure 2 reveal that all the materials exhibited type IV isotherms with H_2_ hysteresis loops. It suggests that cubic mesopores remained intact even after the introduction of NiCo NPs. The SAXRD patterns shown in Figure 3 further confirm the retention of mesoporosity in the CMS support following the inclusion of NiCo NPs. However, the slight decrease in the peak intensities associated with the (110) and (200) crystal planes suggests some deterioration of the mesostructure owing to the occupation of the mesopores by the metal NPs. The powder X-ray diffraction (XRD) patterns of Ni_x_Co_100−x_@CMS (x = 0, 20, 40, 60, 80, 100) are shown in Figure 4. No diffraction peaks corresponding to Co and/or Ni species were observed due to the small particle size and uniform dispersion of metal species. The wide diffraction peaks observed in the 2θ range of 15–35° can be attributed to the amorphous character of the silica. Results from ICP-OES measurements presented in Table 1 reveal that the Ni and Co indeed loaded in the composites. The metal loadings were close to the theoretical loadings of metals, suggesting that the majority of the metal precursors were successfully anchored onto the CMS support.

**Figure 5:**
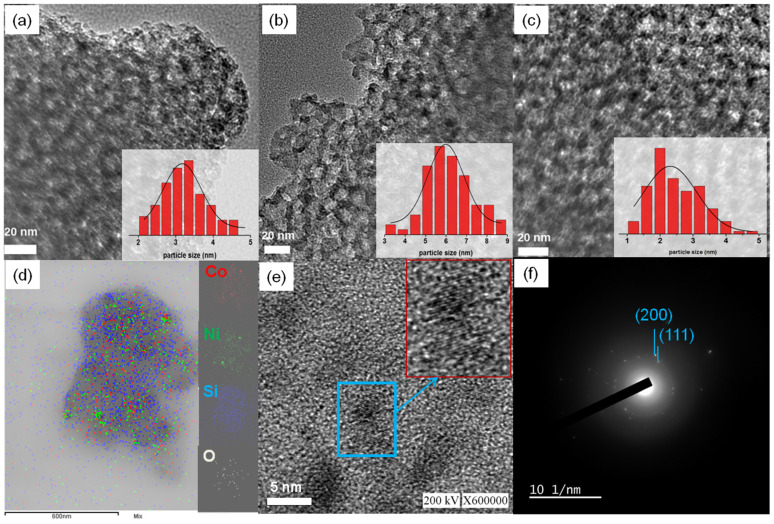
TEM images of (**a**) Co@CMS, (**b**) Ni_60_Co_40_@CMS, and (**c**) Ni@CMS and (**d**) EDS mapping, (**e**) HRTEM image (the enlarge picture of the part enclosed within blue line is shown in the inset) and (**f**) SAED pattern of Ni_40_Co_60_@CMS.

The TEM images of Co@CMS, Ni_40_Co_60_@CMS, and Ni@CMS shown in Figure 5 demonstrate the presence of distinct metal NPs within the pores/on the support surface. The well-ordered arrangements of mesopores in the CMS support were preserved after encapsulation of the metal NPs. The particle size distribution histograms obtained by measuring more than 30 metal NPs, displayed in the insets of Figure 5a,b,c, reveal that the average particle size of the Co, NiCo, and Ni NPs was about 3.2, 2.5, and 6.0 nm, respectively. The development of smaller sized NPs within the channels of the CMS can be attributed to the use of NH_3_BH_3_ as a co-reductant along with NaBH_4_. NH_3_BH_3_ may slow down the reduction rates of both Co^2+^ and Ni^2+^, thereby regulating the growth of the NPs. In the course of synthesizing the NPs, the pH of the solution raised to 9.4, because of the utilization of two reducing agents, NaBH_4_ and NH_3_BH_3_. This led to the de-protonation of both –SiOH and −COOH groups in the CMS to form -SiO^−^ and −COO^−^ groups. Under these circumstances, the strong electrostatic interactions between the negatively charged carboxylate groups and positively charged Co^2+^ and Ni^2+^ ions facilitated easy diffusion of these ions into the mesopores of the CMS, where they were reduced to form NiCo nanoparticles. The strong interaction between the support and the metallic ions is evident from the substantial reduction of the intensity of the band at 1720 cm^−1^ in the FTIR spectra of Ni_40_Co_60_@CMS shown in Figure 1c. As the NPs grew inside the mesopores, the confinement effect prevented the NPs from growing larger than the pore size, leading to formation of highly dispersed small sized NiCo NPs. The smaller sized NPs have a higher surface area and can provide a large number of active sites during the catalysis. The surface modification of the support could enhance the catalytic performance of the NiCo@CMS catalyst by increasing the number of active sites, improving dispersion, and stabilizing the NPs during the reaction. Given that Ni_40_Co_60_@CMS was evaluated as the best catalyst for H_2_ production from AB, more detailed characterizations were conducted for this catalyst. Figure 5d displays a high resolution TEM (HRTEM) image of Ni_40_Co_60_@CMS along with energy-dispersive spectroscopy (EDS) phase mappings. It was observed that Ni and Co elements were dispersed throughout the mesoporous silica support. The HRTEM image shown in Figure 5e exhibits continuous lattice fringes with a *d* spacing of about 0.20 nm, which corresponds to the (111) plane of Ni/Co. The SAED pattern of Ni_40_Co_60_@CMS shown in Figure 5f demonstrates two diffraction rings representative of the (111) and (200) crystal planes of Ni and Co. It indicates that the Co or Ni atoms in the lattice were randomly distributed in the bimetallic NiCo [64].

**Figure 6:**
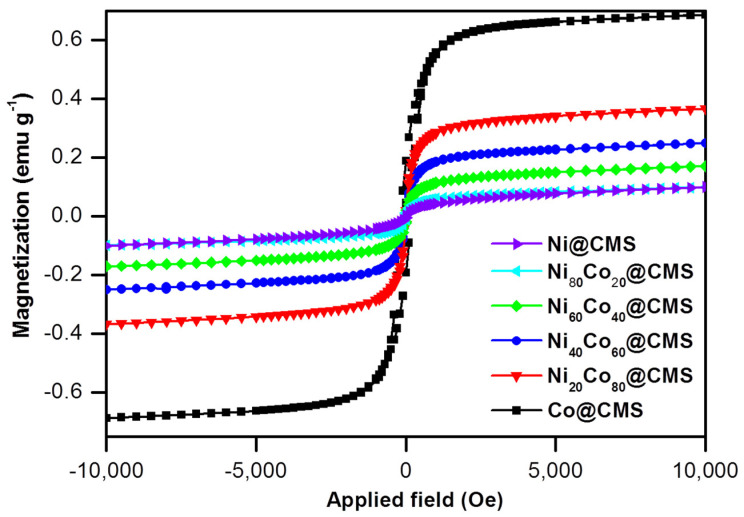
Magnetic hysteresis (M-H) loops of the Ni@CMS, Ni_80_Co_20_@CMS, Ni_60_Co_40_@CMS, Ni_40_Co_60_@CMS, Ni_20_Co_80_@CMS, and Co@CMS at room temperature.

The magnetic properties of the catalysts are essential for their recyclability and reuse. The magnetizations of the Ni_x_Co_100−x_@CMS samples were assessed through the change in magnetization with the variation of the magnetic field, and the outcomes are shown in Figure 6. The hysteresis loops (M-H) recorded via SQUID magnetometry revealed that the NiCo NPs were soft ferromagnetic at room temperature. The saturation magnetization (Ms), coercivity, and remanent values were determined to be 0.64 emu g^−1^ in the presence of 3 kOe of magnetic field and 170 Oe and 0.2 emu g^−1^ for the Co@CMS sample. However, no substantial coercivity and remanent magnetization was detected after the inclusion of Ni, which could be attributed to the lower magnetic moment of Ni (0.6 μB) compared to Co (1.72 μB) [65].

**Figure 7:**
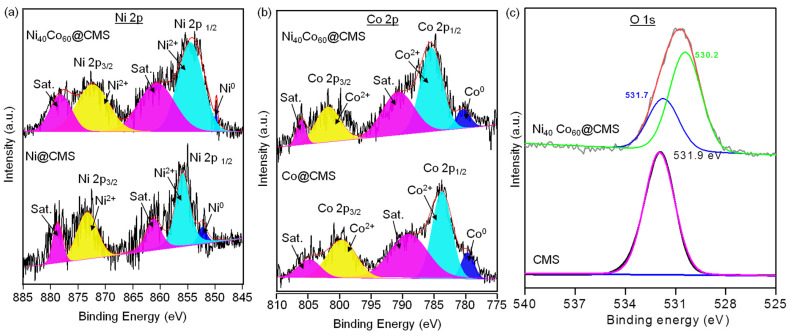
High-resolution XPS spectra of (**a**) Ni 2p for Ni@CMS and Ni_40_Co_60_@CMS, (**b**) Co 2p for Co@CMS and Ni_40_Co_60_@CMS and (**c**) O 1s spectra for CMS and Ni_40_Co_60_@CMS.

The XPS analyses of CMS and Ni_40_Co_60_@CMS were performed to study their electronic structure and chemical states. The XPS survey spectra of the CMS shown in Figure S2 include bands corresponding to the core levels of Si 2p (99–104 eV), Si 2s (150–155 eV), O 1s (530–533 eV), and C 1s (285 eV) [66]. The deconvoluted XPS spectra of Si 2p showed two peaks at 102 and 103.5 eV that confirmed the presence of SiOH and SiO_2_. The shifting of the O 1s binding energy of CMS to 531.9 eV could be attributed to the change in the electron density and bonding environment after functionalization. High-resolution XPS was performed to examine the chemical states of the Ni, Co, and bimetallic NiCo NPs. Figure 7a shows the high-resolution XPS spectra of Ni 2p of the monometallic Ni@CMS and bimetallic Ni_40_Co_60_@CMS. The spectrum showed two pairs of doublets at about 859 and 876 eV, which could be assigned to Ni 2p_3/2_ and Ni 2p_1/2_ states of metallic Ni. The deconvolution of the spectrum of Ni@CMS showed a peak at 852.2 eV representative of zero-valent Ni, whereas the other peaks at 855.8 and 873.2 eV were due to the oxidized Ni [44]. Moreover, two shake-up satellites of Ni^0^ at 861.1 and 878.6 eV were also observed. The peak position shifted towards the lower binding energy side for the Ni_40_Co_60_@CMS. As for the high-resolution Co 2p spectrum of Co@CMS shown in Figure 7b, the binding energies of 783 and 800 eV can be assigned to 2p_3/2_ and 2p_1/2_, revealing that the Co species mainly existed in the oxide state [44]. In comparison to Co@CMS, the binding energy in the Co 2p region of Ni_40_Co_60_@CMS shifted to the higher binding energy side by 0.6 eV. The intensities corresponding to the oxides species were significantly higher than those of Ni^0^ and Co^0^, indicating that Ni^2+^ and Co^2+^ were the dominant states after exposure to oxygen in the air. The opposite variation in binding energy shifting of Ni 2p and Co 2p indicates that some electrons may be transferred from Ni to Co atoms, confirming the interaction between the Ni and Co atoms and the formation of bimetallic NiCo nanoparticles. The deconvoluted O 1s XPS spectrum presented in Figure 7c shows the peaks at 530.2 eV and 531.7 eV, which confirms the presence of metallic oxide (NiO, CoO) and hydroxide, respectively [67]. The shift in the O 1s binding energy from 531.9 eV, the binding energy characteristics of CMS, evidenced the interactions between the support and the metal species. These changes suggest the formation of new chemical bonds, such as Ni-O-Si or Co-O-Si, confirming the metal–support interaction.

### 3.2. Catalytic Hydrolysis of Ammonia Borane by Ni_x_Co_100−x_@CMS for Hydrogen Generation

**Figure 8:**
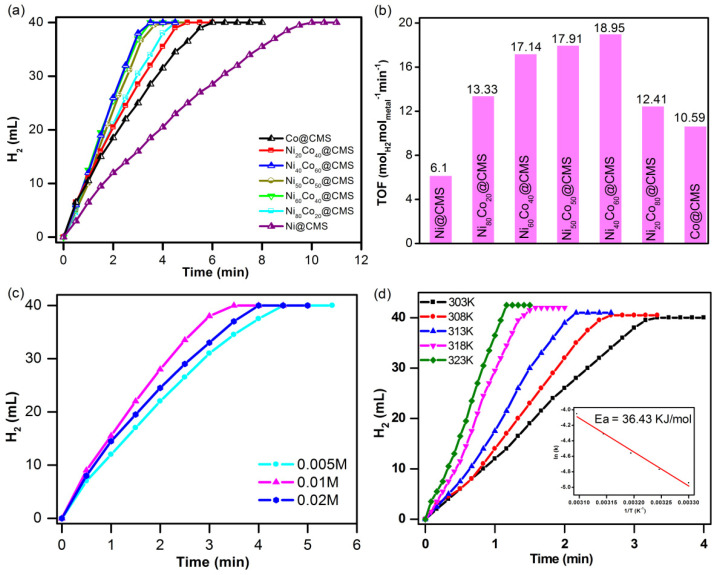
Plots of time versus volume of H_2_ generation from hydrolysis of ammonia borane by (**a**) Ni@CMS, Ni_80_Co_20_@CMS, Ni_60_Co_40_@CMS, Ni_50_Co_50_@CMS, Ni_40_Co_60_@CMS, Ni_20_Co_80_@CMS, and Co@CMS and (**b**) the corresponding TOF values, (**c**) H_2_ generation by Ni_40_Co_60_@CMS at different metal concentrations, and (**d**) H_2_ generation by Ni_40_Co_60_@CMS at different temperatures. The Arrhenius plot obtained from the reaction rate constant (k) at different concentrations is shown in the inset.

The catalytic activities of the prepared Ni_x_Co_100−x_@CMS materials were explored by using them as catalysts for AB hydrolysis. Figure 8a shows the H_2_ generation by Ni_x_Co_100−x_@CMS during the hydrolytic dehydration of NH_3_BH_3_ over time. It was revealed that all the metal catalysts were able to generate a stoichiometric amount of H_2_ (40 mL) from hydrolysis of AB. The steady rise in the volume of H_2_ generated over time during the early stage of the hydrolysis reaction suggests a zero-order reaction, implying that the reaction was not limited by external diffusion of the reactant. As observed in Figure 8a, under identical reaction conditions, monometallic Ni@CMS produced H_2_ at a significantly slower rate compared to Co@CMS. However, with the addition of Co to Ni while maintaining the same total moles of (Ni + Co) in the catalyst intact, the reaction time for complete hydrolysis of AB reduced substantially compared to those of Ni@CMS and Co@CMS. The reaction rate constants (*k*) were calculated from the linear parts of the plots, and the estimated rates were 2.6, 5.7, 7.3, 7.6, 8.0, 5.2, and 4.5 L min^−1^ g^−1^ for Ni@CMS, Ni_80_Co_20_@CMS, Ni_60_Co_40_@CMS, Ni_50_Co_50_@CMS Ni_40_Co_60_@CMS, Ni_20_Co_80_@CMS, and Co@CMS, respectively. The optimal reaction rate of 8.0 L min^−1^ g^−1^ was accomplished at the Ni to Co ratio of 40 to 60. The turnover frequencies (TOFs) of the catalysts were determined using the following relationship,

(1) $$TOF=\frac{V_{H_{2}}}{22.4V_{s}C_{Catalyst}t}$$

where V_H2_ is the volume of *H*_2_ generated, vs. the solution volume, *C_Catalyst_* the concentration of catalyst, and *t* the time for the reaction to finish. The estimated TOF values of the catalysts are shown in Figure 8b, which clearly show a volcano type activity trend, i.e., the TOF value increases with the increase in the Co content in the NiCo alloy and reaches a maximum TOF of 18.95 mol_H2_min^−1^mol_Catalyst_^−1^ with Ni_40_Co_60_@CMS. The increase in H_2_ generation using bimetallic Ni_x_Co_100−x_@CMS (x = 20, 40, 50, 60, and 80) catalysts could be attributed to the combination of several factors such as the redox reaction involved during the hydrolysis, synergistic cooperation between Co and Ni, tuned electronic structures, and catalytic activation. The XPS analysis showed the presence of NiO, which could reduce the CoO during the NH_3_BH_3_ hydrolysis through a redox reduction process. In the reaction, NiO acted as the reducing agent and CoO as an oxidizing agent. NiO could donate electrons to CoO, thereby reducing CoO to Co metal [68], which can act as an additional catalyst and improve the overall reaction kinetics and H_2_ generation rate. Another reason for enhanced catalytic activity could be the synergistic effects between the Ni and Co. The interactions between Ni and Co atoms in bimetallic NPs can modify the electronic structure, resulting in an optimized distribution of electron density [35]. It can improve the adsorption and activation of NH_3_BH_3_ molecules on the catalyst surface, making the hydrolysis reaction more efficient. In bimetallic NiCo, both Ni and Co atoms are present on the surface, creating dual active sites. These active sites work together more efficiently than either metal alone to facilitate the cleavage of N-H and B-H bonds.

**Scheme 1:**
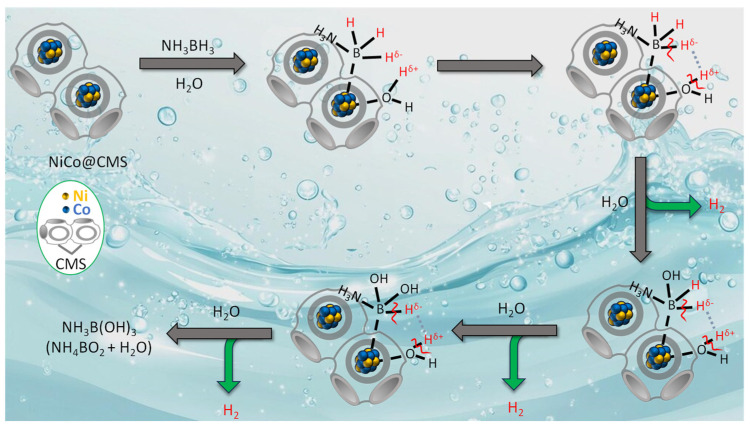
Schematic illustration of the metal catalyzed hydrolysis of AB for hydrogen generation.

The best activity of Ni_40_Co_60_@CMS among all the composites investigated could be attributed to the electron transfer from Ni to Co, which might have finely tuned the electronic structure and created an optimized d-band center [35]. The finely tuned electronic structure could enhance the adsorption of AB, activate the hydrogen bonds, and subsequently cause faster AB hydrolysis. The catalytic activity of the parent CMS support was also tested to assess the role in influencing the catalytic performance of the nanocomposite. Figure S3 illustrates the amount of H_2_ generated by the CMS over time, revealing that CMS without metal NPs is unable to fully catalyze NH_3_BH_3_. The reaction progressed at a much slower rate compared to those involving the metallic components. The insignificant catalytic activity of the CMS was due to the acid-catalyzed hydrolysis of NH_3_BH_3_, likely caused by the deprotonation of the carboxylic acid groups, leading to the production of hydronium ions. Although CMS exhibited insignificant activity towards the AB hydrolysis, its mesopores functioned as nanoreactors to promote the smooth transportation of reactants and products. The catalytic activities of various Ni-based catalysts reported in the literature presented in Table 2 suggests that the catalytic performance of Ni_40_Co_60_@CMS was better or comparable to most of the NiCo-based catalysts reported in the literature for the same reaction. The variations in the catalytic activities of NiCo catalysts encapsulated within different supports could be attributed to the support’s unique textural properties, distinct surface chemistries, and metal–support interactions. H_2_ generation from the catalytic hydrolysis of AB using Ni@CMS, Co@CMS, and Ni_x_Co_100−x_@CMS as the catalyst involves a series of steps such as (i) the formation of an activated complex between AB and the metal surface, (ii) cleavage of the B–N bond assisted by a H_2_O attack, and (iii) hydrolysis of the resulting BH_3_ moiety to form H_2_ and BO_2_^−^ [69]. The process begins with the adsorption of NH_3_BH_3_ molecules on the surface of the catalyst, which provides active sites for adsorption. Upon adsorption, the N-H and B-H bonds in NH_3_BH_3_ are weakened due to the interactions between the electron-rich metal atoms and electron-deficient boron atoms, as well as the interactions between the nitrogen atom and the metal surface. The weakening of the B-H bonds can lead to the formation of metal-hydride (M-H) or metal boron (M-B) intermediates. Water molecules, which are present in the reaction medium, also adsorb onto the catalyst surface. The hydrolysis reaction involves the cleavage of the water molecules into H^+^ and OH^−^. The produced OH^−^ interacts with the M-H intermediates and promotes the release of H_2_. Simultaneously, the N-H bonds may also be broken to contribute additional H_2_ release. The proper adsorption of H_2_O molecules by the catalyst is therefore highly important as the –OH species formed due to the decomposition of H_2_O molecules attack the B-N bond in ammonia borane to produce H_2_. During the hydrolysis of ammonia borane by Ni_x_Co_100−x_@CMS, the presence of surface oxygen vacancies on the catalyst support, as evidenced from the peak at 531.7 eV in the deconvoluted O 1s XPS spectrum (Figure 7) [70,71] eased the adsorption of H_2_O molecules. The oxygen vacancies introduced local defects in the silica lattice, which served as the electron donors. The increase in electron density stabilized the NiCo NPs, modified their electronic structure, and facilitated better electron transfer to ammonia borane molecules and promoted rapid cleavage of H bond. In addition, the presence of -COOH functional groups on the CMS support made the surface of the Ni_x_Co_100−x_@CMS nanocatalyst more hydrophilic. The enhanced hydrophilicity ensured high dispersion of the catalyst in the reaction solution. The homogeneous dispersion of the catalyst was advantageous as it increased the opportunities for interfacial contact between the Ni-Co sites and ammonia borane molecules during hydrolysis. The efficient contact between the catalyst and reactant lead to the significant enhancement in catalytic activity of NiCo@CMS for ammonia borane hydrolysis. XPS measurements (Figure 7) confirmed that there was electron transfer from Ni to Co atoms, which would lead to a change in the local charge density of the metal surface. Moreover, the metal–support interaction between NiCo nanoparticles and CMS promoted the electrons transfer from Ni to support and support to Co atoms, further enhancing the change of charge density of the metal surface (Figure 7). Therefore, as illustrated in Scheme 1, which represents the plausible ammonia borane hydrolysis mechanism catalyzed by Ni_x_Co_100−x_@CMS, the NiCo dual site has a strong electrostatic interaction with AB molecules, leading to better catalytic activity. The process starts with the adsorption of H_2_O and NH_3_BH_3_ molecules onto the catalyst surface. Due to the difference in electronegativity, the hydrogen atom in H-OH of H_2_O is protic, whereas the hydrogen in the B-H bond of AB is hydridic. Upon adsorption of AB on the metal NPs, the B-H and N-H bonds in AB were weakened. The H-OH and B-H bonds were cleaved, and the generated H^δ+^ combines with H^δ−^ to produce molecular H_2_. At the same time, the remaining -OH combined with -BH_2_ to produce the intermediate product NH_3_BH_2_OH. Subsequently, the H-OH bond from the second adsorbed H_2_O molecule and the B-H bond from the NH_3_BH_2_OH were further broken, resulting in the formation of the second molecular H_2_ and the intermediate product NH_3_BH(OH)_2_. Next, the H-OH bond from the third adsorbed H_2_O molecule and the B-H bond from NH_3_BH(OH)_2_ were broken, producing another H_2_ molecule and the intermediate product NH_3_B(OH)_3_, which ultimately decomposed to NH_3_BO_2_ and H_2_O. The different reaction steps occurred can be summarized as

(2) $$NH_{3}BH_{3}+2M⇄NH_{3}BH_{2}M+MH(M\to {\mathrm{Ni}}_{x}{\mathrm{Co}}_{100-x})$$

(3) $$NH_{3}BH_{2}M+H_{2}O\to NH_{3}BH_{2}OH+MH$$

(4) $$NH_{3}BH_{2}OH+2M\to NH_{3}BH\left(OH\right)M+MH$$

(5) $$NH_{3}BH\left(OH\right)M+H_{2}O\to NH_{3}BH{\left(OH\right)}_{2}+MH$$

(6) $$NH_{3}BH{\left(OH\right)}_{2}+2M\to NH_{3}B{\left(OH\right)}_{2}M+MH$$

(7) $$NH_{3}B{\left(OH\right)}_{2}M+H_{2}O\to NH_{3}B{\left(OH\right)}_{3}+MH$$

(8) $$MH+MH\to 2M+H_{2}$$

(9) $$NH_{3}B{\left(OH\right)}_{3}\to NH_{4}^{+}+BO_{2}^{-}+H_{2}O$$

The kinetics of AB hydrolysis was investigated by keeping the catalyst to ammonia borane concentration constant at 0.05 M and varying the Ni_40_Co_60_@CMS catalyst concentration from 0.005 to 0.02 M, and the effect is displayed in Figure 8c. The time needed for hydrolysis of the whole AB shortened from 4.5 to 3.5 min on increasing the catalyst concentration from 0.005 to 0.01 M, which could be attributed to the increase in the active site as the catalyst concentration rises. However, the time required for the complete hydrolysis of AB extended to 4 min at the catalyst concentration of 0.02 M, which could be attributed to the formation of larger sized NPs at high metal concentrations. This reduced the surface area available for the catalysis and eventually decreased the hydrolysis rate. The influence of the reaction temperature on the H_2_ generation rate is shown in Figure 8d. It was observed that the reaction time shortened notably from 4 to 1.16 min when the reaction temperature was increased from 303 to 323 K. The reaction rate constants (*k*) at various temperatures were estimated from the slope of the linear section of each plot. The activation energy (E_a_) was estimated from the plot of ln(k) versus 1/T (inset of Figure 8d) using the Arrhenius equation,

(10) $$\ln k=-\frac{E_{a}}{RT}+lnA$$

where R is the ideal gas constant, A is the pre-exponential factor, and T is the reaction temperature in Kelvin. The E_a_ value, which reflects the minimum energy required for the occurrence of a chemical reaction, was determined as 36.43 kJ mol^−1^ for Ni_40_Co_60_@CMS, for the complete hydrolysis of AB. In general, a higher catalytic activity corresponds to a lower activation energy value. The lower or comparable E_a_ value relative to previously reported NiCo-based catalysts presented in Table 2 further confirmed the high efficiency of the Ni_40_Co_60_@CMS catalyst for the AB hydrolysis. It can therefore be concluded that the energy barrier can be substantially reduced by optimizing the composition of NiCo alloy catalysts.

**Figure 9:**
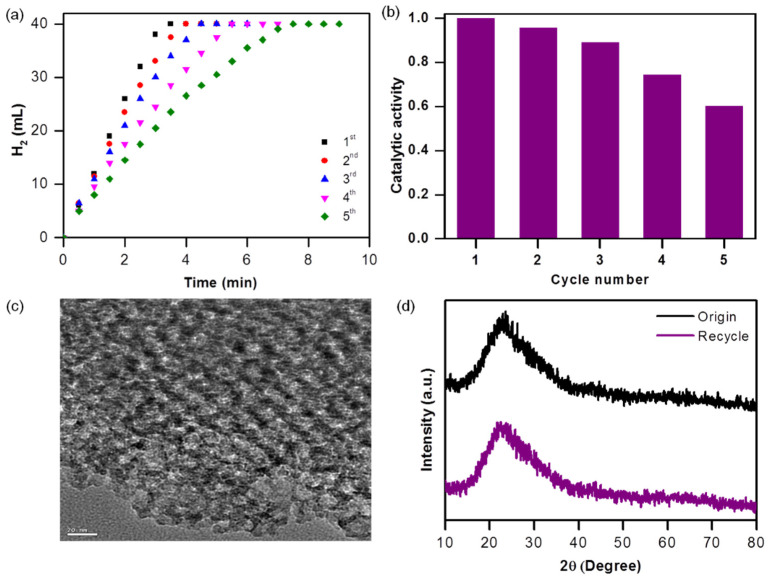
(**a**) Plot of time versus H_2_ generation from AB hydrolysis Ni_40_Co_60_@CMS catalyst from the 1st to 5th cycles, (**b**) catalytic activity versus cycle number, (**c**) TEM image, and (**d**) WAXRD pattern of the recycled Ni_40_Co_60_@CMS.

**Figure 10:**
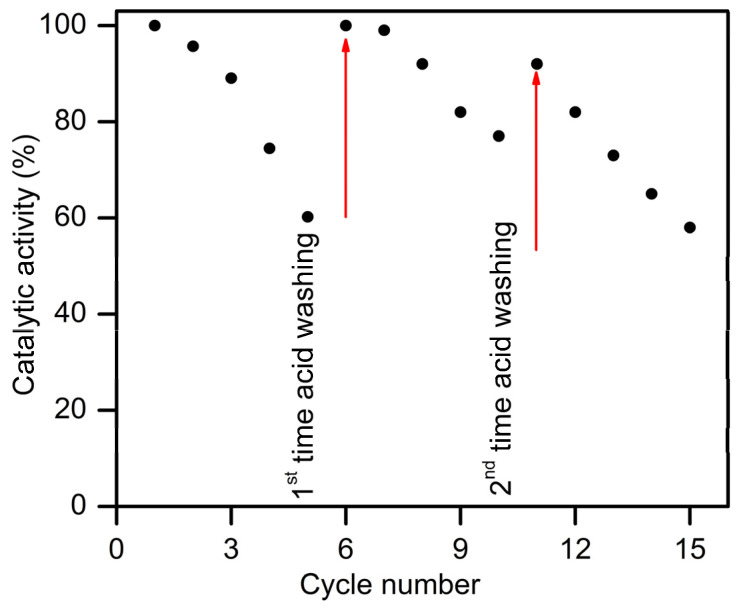
Catalytic activity of regenerated Ni_40_Co_60_@CMS catalyst as a function of cycle number for ammonia borane hydrolysis.

A consecutive recycle test was carried out to investigate the durability of the Ni_40_Co_60_@CMS catalyst. As shown in Figure 9a, the catalyst was able to produce a stoichiometric amount of H_2_ even after five cycles, although the reaction rate declined with each successive cycle. It was observed that about 90% (Figure 9b) of the catalytic activity of its initial state was preserved after being used for three consecutive cycles, suggesting the excellent durability of the present nanocatalyst. The outstanding reusability is likely due to the strong metal–support interactions facilitated by the carboxylate group in the support. This interaction helps to anchor the NiCo NPs securely within the well-ordered mesopores of the CMS, preventing both aggregation and leakage, thus preserving the active sites. The TEM image of the Ni_40_Co_60_@CMS catalyst after the durability test displayed in Figure 9c confirms the well-preserved morphology of the Ni_40_Co_60_@CMS catalyst after catalytic cycles. However, the particle size increased slightly from 2.50 to 3.41 nm after being used for 5 cycles, which could be one of the reasons for the reduced activity to 60% for the 5th cycle. The recycled catalysts were further analyzed via WAXRD analysis, and the XRD pattern of the recycled Ni_40_Co_60_@CMS catalyst is presented in Figure 9d. The XRD pattern was similar to that of the fresh catalyst, indicating that there were no significant changes in the microstructural properties after multiple cycles of use. The drop in activity after being used for multiple cycles could be linked to the loss of certain catalysts during washing over time. In addition, the repeated use of the catalyst might lead to the adsorption of metaborate ions on its surface [72], which hindered the access to the NiCo active sites, potentially contributing to the reduced catalytic performance. After the 5th cycles, the used catalyst was separated from the spent solution and washed with dilute acid (0.001M HCl) to regenerate the catalyst and regain its activity. The acid wash can remove the B-O-based species accumulated after each use and has proven to be an efficient deactivation mechanism for Co-based catalysts for this reaction [73]. After cleaning with acid, the catalyst was washed with distilled water to remove any impurity that may present on the catalyst surface. NH_3_BH_3_ was then added to the reactor to initiate the next cycle. The activity versus cycle number plot of the regenerated catalyst shown in Figure 10 demonstrates that the catalytic activity recovered significantly after 5 cycles. It indicates that acid washing was successful in removing the borate ions largely. However, the catalytic activity decreased after 10 cycles and reached about 50% of the initial value after 15 cycles. Further investigation needs to be conducted in the near future to boost the durability using different solvents including acetone, methanol, bases, etc.

As both the metallic components, Ni and Co, in Ni_x_Co_100−x_@CMS are naturally abundant and inexpensive, it is a cost-effective choice for AB hydrolysis to generate H_2_ as compared to other bimetallic nanocatalysts that rely on costly precious metals such as Pt, Pd, or Ru as one of their components. Additionally, the catalysts showed superb reusability and durability over multiple cycles. As a result, there was no need for frequent replacement of the catalyst during AB hydrolysis. It can thus minimize waste generation as well as conserve natural resources. On the other hand, non-metallic catalysts, such as carbon-based or organic catalysts, often degrade faster, which leads to higher waste and loss of resources over time. Furthermore, both Ni and Co have relatively low toxicity compared to bimetallic catalysts involving toxic heavy metals, such as Cd-based catalysts AuCd, CuPb, NiCd, etc. Thus, use of NiCo@CMS as a catalyst could minimize the potential environmental hazards associated with catalyst disposal, signifying its environmental sustainability. The efficient H_2_ release, low cost, and good stability could make Ni_x_Co_100−x_@CMS a superior choice for various hydrogen-based applications, including portable power sources, small-scale fuel cell systems, etc.

## 4. Conclusions

In this study, bimetallic NiCo NPs were successfully immobilized within the mesopores of –COOH functionalized mesoporous silica and utilized for the hydrolysis of AB to produce H_2_. The organic functional groups in the support facilitated a uniform dispersion of the metal NPs, while the ordered mesopores regulated the growth of the metal NPs. The bimetallic NiCo NPs demonstrated significantly higher catalytic activity for hydrolysis of AB compared to their monometallic counterparts. The superior activity was attributed to several factors, including the redox reaction during hydrolysis, synergistic effects between Ni and Co, modified electronic structures, and enhanced catalytic activation. However, the catalytic performance of the bimetallic nanocomposite was composition-dependent. Of all the compositions, Ni_40_Co_60_@CMS exhibited the highest catalytic activity, achieving a TOF of 18.95 mol_H2_min^−1^mol_Catalyst_^−1^ and a hydrogen generation rate of 8.0 L min^−1^ g^−1^. This was due to the finely-tuned electronic structure resulting from electron transfer from Ni to Co. While the catalyst exhibited excellent catalytic performance in the laboratory scale, it is essential to ensure it can withstand industrial conditions, such as high temperature, extended operation times, and adaptability to various reactor designs. Although reusability of the catalyst could be significantly enhanced via reactivation and regeneration, further enhancement is necessary for industrial applications. This could be achieved by using stabilizing agents, doping with additional metals, incorporating metal oxide prompters, or functionalizing mesoporous silica with other functional groups such as sulfonic acid, amine, etc. The synthesis process needs to be optimized from lab-scale co-precipitation to a large-scale impregnation method to produce high quality catalysts in large quantities. Biomass-derived porous carbons can be used as alternative supports to immobilize metal NPs with consideration of cost-effective synthesis methods to ensure economic viability at a large scale. The application possibilities of the synthesized NiCo@CMS will be further explored regarding its hydrogen evolution reaction in water electrolysis. The catalysts also have tremendous potential for conversion of biomass into biofuels via processes such as gasification and pyrolysis.

## Figures and Tables

**Table 1:** Textural properties of bimetallic Ni_x_Co_100−x_@CMS and composition of metals.

Sample	Surface Area (m^2^ g^−1^)	Pore Volume (cm^3^ g^−1^)	Pore Size (nm)	Metal Amount (wt.%) ^a^	
CMS	616	1.16	6.0	-	
Ni@CMS	450	1.05	6.0	6.11	Ni (6.11)
					Co (-)
Co@CMS	468	1.05	6.0	4.95	Ni (-)
					Co (4.95)
Ni_80_Co_20_@CMS	444	1.04	6.0	4.87	Ni (3.84)
					Co (1.03)
Ni_60_Co_40_@CMS	435	1.03	5.8	5.07	Ni (2.07)
					Co (3.00)
Ni_40_Co_60_@CMS	490	1.09	5.9	5.83	Ni (2.22)
					Co (3.61)
Ni_20_Co_80_@CMS	454	1.05	6.0	5.90	Ni (1.13)
					Co (4.77)

^a^ Amount of metal loading determined via ICP-AES.

**Table 2:** H_2_ production from AB hydrolysis by bimetallic NiCo NPs encapsulated within various supports.

Catalysts	Temperature (°C)	TOF (mol_H2_min^−1^mol_Catalyst_^−1^)	E_a_ (kJ mol^−1^)	Ref.
Ni_40_Co_60_@CMS	30	18.95	36.43	This work
NiCo-NC	25	16.7	43.6	[33]
CoNi/MCNTs	30	33	52.1	[49]
CoNi@RGO	25	19.54	39.89	[53]
NiCo-GO	25	6.78	-	[54]
CoNi/XC-72	50	49	28.9	[55]
CoNi/Al_2_O_3_	25	34.5	32.9	[56]
CoNi@h-BN	20	-	28	[57]
Co_89.8_Ni_10.8_P_11.7_/rGO	25	18.6	25.0	[58]
Co_3.33_Ni_1.67_/SAPO-34-N	30	9.25	55.01	[59]

## Data Availability

Data are contained within the article and Supplementary Materials.

## Supplementary Materials

The following item is available online at https://www.mdpi.com/article/10.3390/nano14221818/s1, Figure S1. (a) Acid-base titration and (b) zeta potential plots of Ni_40_Co_60_@CMS at different pH; Figure S2. XPS survey, Si 2p and O 1s spectra of CMS; Figure S3. H2 generation from NH_3_BH_3_ hydrolysis by CMS.
