# Supplementary material for: Bimetallic NiCo Nanoparticles Embedded in Organic Group Functionalized Mesoporous Silica for Efficient Hydrogen Production from Ammonia Borane Hydrolysis
Source: Nanomaterials (Basel). 2024 Nov 13;14(22):1818. doi: 10.3390/nano14221818 (PMC11597744; doi:10.3390/nano14221818)
Supplement: Supplementary file 1 [file nanomaterials-14-01818-s001.zip › nanomaterials-3270256-supplementary.pdf]

Supplementary Information

Bimetallic NiCo Nanoparticles Embedded in Organic Group Functionalized Mesoporous Silica for Efficient Hydrogen Production from Ammonia Borane Hydrolysis

Juti Rani Deka [1], Diganta Saikia [2], Ning-Fang Lu [2], Chieh-Yu Chen [2], Hsien-Ming Kao [2,*] and Yung-Chin Yang [1,*]

[1] Institute of Materials Science and Engineering, National Taipei University of Technology, Taipei 106344, Taiwan

[2] Department of Chemistry, National Central University, Zhongli 320317, Taiwan

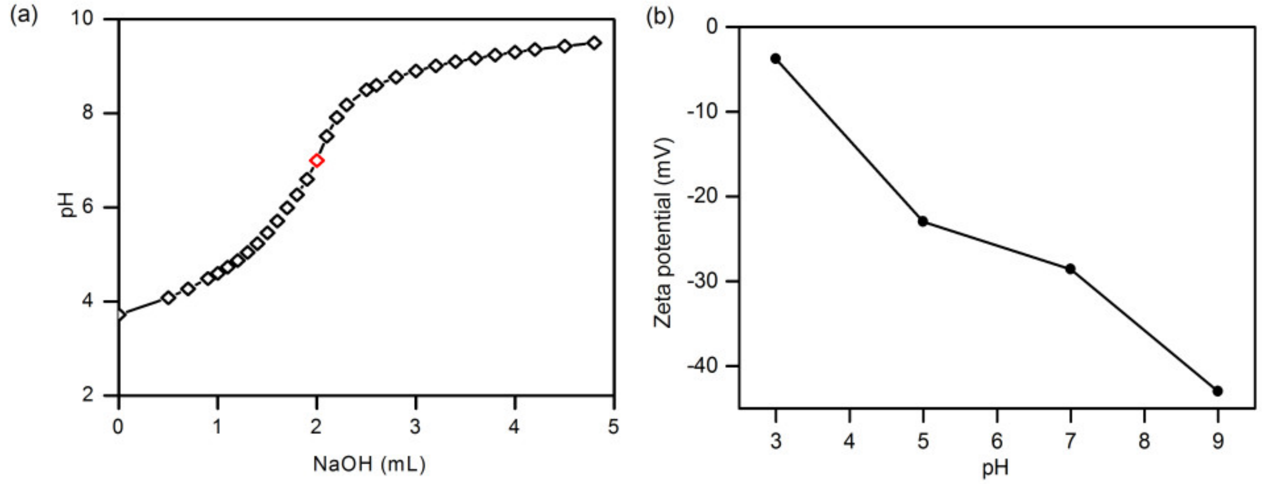

Figure S1. (a) Acid-base titration and (b) zeta potential plots of $Ni_{40}Co_{60}$@CMS at different pH.

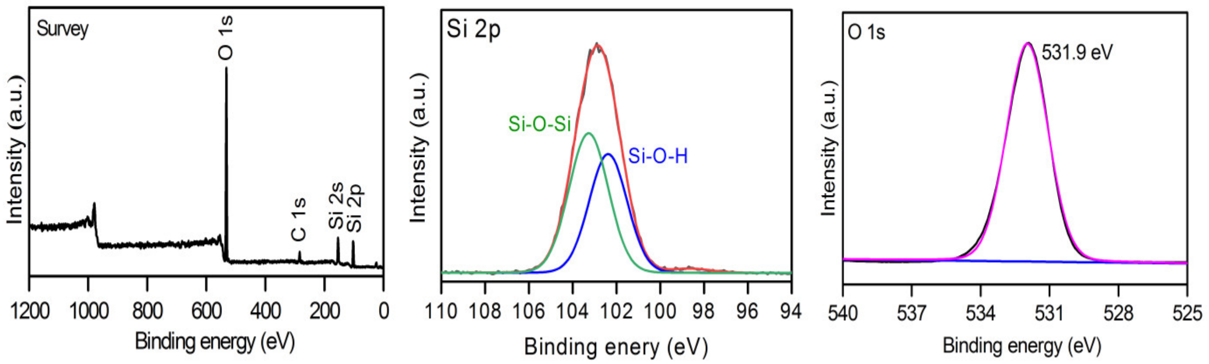

Figure S2. XPS survey, Si 2p and O 1s spectra of CMS.

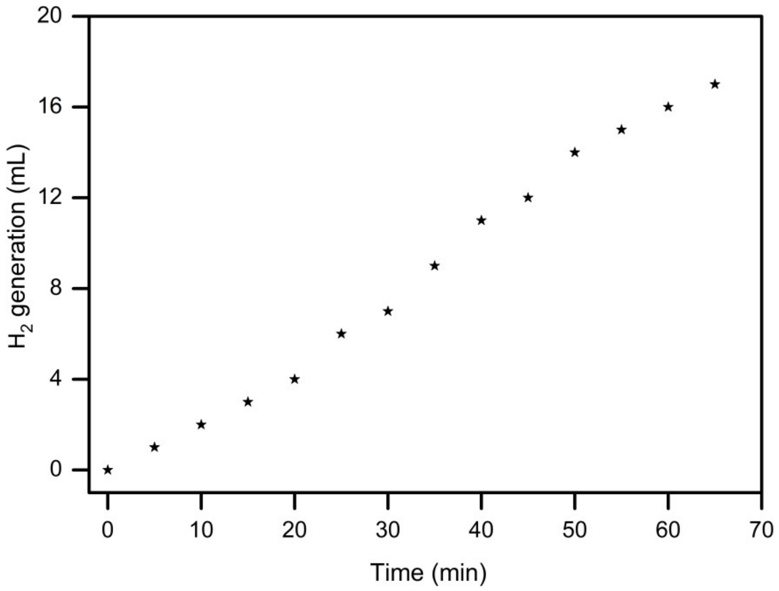

Figure S3. H_2 generation from NH_3BH_3 hydrolysis by CMS.
